# A Bayesian Assessment of an Approximate Model for Unconfined Water Flow in Sloping Layered Porous Media

**DOI:** 10.1007/s11242-018-1094-2

**Published:** 2018-06-09

**Authors:** Juan Chiachío, Manuel Chiachío, Shankar Sankararaman, Darren Prescott

**Affiliations:** 1 Resilience Engineering Research Group, University of Nottingham, Nottingham, UK; 2 SGT Inc., NASA Ames Research Center, Moffett Field, CA 94035-1000 USA

**Keywords:** Dupuit–Forchheimer analysis, Layered porous media, Bayesian hypothesis testing, Railway track drainage

## Abstract

The prediction of water table height in unconfined layered porous media is a difficult modelling problem that typically requires numerical simulation. This paper proposes an analytical model to approximate the exact solution based on a steady-state Dupuit–Forchheimer analysis. The key contribution in relation to a similar model in the literature relies in the ability of the proposed model to consider more than two layers with different thicknesses and slopes, so that the existing model becomes a special case of the proposed model herein. In addition, a model assessment methodology based on the Bayesian inverse problem is proposed to efficiently identify the values of the physical parameters for which the proposed model is accurate when compared against a reference model given by MODFLOW-NWT, the open-source finite-difference code by the U.S. Geological Survey. Based on numerical results for a representative case study, the ratio of vertical recharge rate to hydraulic conductivity emerges as a key parameter in terms of model accuracy so that, when appropriately bounded, both the proposed model and MODFLOW-NWT provide almost identical results.

## Introduction

The modelling of unconfined water flow in layered porous media is a challenging problem with important applications in Earth sciences and engineering. Relevant examples of such applications are found in the drainage of agricultural lands (Schmid and Luthin 1964), or the internal drainage of ballasted railway tracks (Rushton and Ghataora 2009), among others. An exact analytical solution to the problem is virtually impossible due to the nonlinearity of the unconfined boundary condition and the fact that the location of this boundary is unknown (Bear 1972). This modelling complexity is accentuated when dealing with sloping layered porous media with recharge (Rushton and Youngs 2010), which typically requires the use of numerical methods such as finite-difference (FD) (Wang and Anderson 1982; Todsen 1971; Lee and Leap 1997) or finite element (FE) models (Shamsai and Narasimhan 1991; Rulon et al. 1985; Chen et al. 2008; Zheng et al. 2009) to approximate the exact solution. However, these numerical methods become computationally demanding and hence non-feasible for several activities such as model calibration, parameter estimation and optimization, since such analyses require a great number of model evaluations. Groundwater numerical models, especially those considering unconfined flow with recharge (e.g. precipitation), may require significant CPU time to complete a single forward run.  Approximate models can be used to address the computational complexity of the numerical models; however, they require a number of simplifying assumptions so that they can be solved analytically.

The Dupuit–Forchheimer (D–F) theory is perhaps the most powerful and widely accepted simplifying theory for treating unconfined flows (Bear 1972), although most of the available solutions are restricted to homogeneous isotropic porous media (Schmid and Luthin 1964; Wooding and Chapman 1966; Towner 1975; Chapman 1980; Yates et al. 1985; Knight 2005; Castro-Orgaz and Giráldez 2012). Despite its practical relevance, very few references can be found in the literature dealing with some form of approximate model to efficiently approach the problem of unconfined water flow in layered porous media with recharge. Youngs (1965, 1966) provided an analytical formulation of the unconfined seepage flow problem in soils with hydraulic conductivity varying with depth that was further extended in Youngs (1971) for sloping lands; notwithstanding, these works do not consider solutions for water table profiles. More recently, Youngs and Rushton (2009b) have provided an approximate model for steady-state water table prediction in two-layered undulating soils with recharge in the context of a railway track drainage problem, although it is restricted to systems with two parallel layers, which significantly bounds the practical scope of the proposed solution. Moreover, there are known limitations of the D–F theory based on the assumed simplifying hypotheses about seepage flow. Most authors agree that solutions must be restricted to problems where flow is essentially horizontal with a small inclination of the water table (Bear 1972; Lee and Leap 1997; Castro-Orgaz and Giráldez 2012). Others, in contrast, have shown that D–F solutions are sufficiently accurate even when water table slope is considerable and there is a significant vertical velocity component (Towner 1975; Youngs and Rushton 2009a, b). However, as evident from the results in Youngs and Rushton (2009a, b), the accuracy of the proposed D–F approximations greatly depends on the adopted values of some model parameters. These model parameters are not fitting parameters that need to be tuned nor estimated by comparing model predictions against observed data. Rather, these are physical parameters that represent the actual properties of the porous medium. Hence, it is important to identify the values of the model parameters that makes the D–F approximation accurate when compared against a system response taken as a benchmark.

**Fig. 1:**
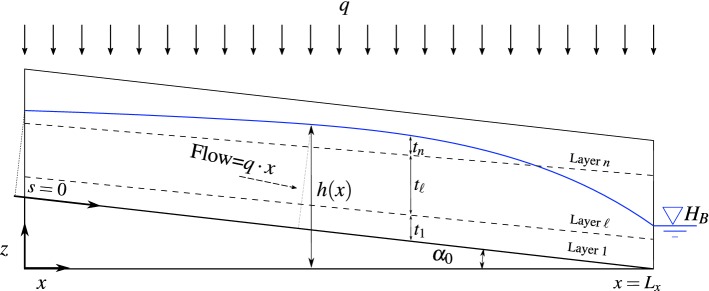
Steady-state unconfined water table representation (blue solid line) for a 2D *n*-layered sloping porous medium. The impervious base is represented in grey colour, and the boundaries between layers with different hydraulic conductivities are represented by dashed lines

In this context, the contribution of this paper is threefold: First, an approximate model for steady-state water flow in multilayered porous media with recharge is presented based on the D–F theory. The model predicts the water table elevation for unconfined sloping systems with an unlimited number of non-parallel layers, where the recharge is drained to a downstream boundary. See Fig. 1 for a schematic representation of the system considered. After solving the resulting differential equation, the solution obtained for water table height is shown to generalize the one proposed by Youngs and Rushton (2009b) for two parallel layers, so that it becomes a particular case of the model proposed herein. In addition, an efficient approach based on the Newton–Raphson method is proposed to accurately determine the crossing points where water table intersects the interface of layers with contrasting hydraulic conductivities, which is a known difficulty when dealing with layered porous media (Youngs and Rushton 2009b; Rushton and Youngs 2010). The method is intended to prevent inaccuracies in the solution due to uncontrollable error propagation.

Second, this paper proposes a Bayesian inverse problem methodology (Tarantola 2005; Rus et al. 2016) that identifies the values of the parameters for which the proposed model is more likely to perform identical to a reference numerical model using MODFLOW (Harbaugh 2005). MODFLOW is the open-source FD model by the U.S. Geological Survey and the most widely used computer code to solve the exact formulation of the problem of water flow in porous media. By the proposed methodology, the identification of model parameters is formulated as a probabilistic inverse problem within the framework of Bayesian hypothesis testing, since it provides a rigorous framework to account for the various types of modelling uncertainties (e.g. discretization error and truncation error) within the assessment. The *null hypothesis* in this inference problem corresponds to the event that the hypothesized model is equivalent to the reference MODFLOW model. Relative probabilities are used to quantify the degree of belief that the null hypothesis is true, conditioned on the values of model parameters. Next, an inverse problem is formulated based on Bayes’ theorem where the probability distributions of the model parameters are estimated conditioned on the event that the null hypothesis holds.

**Fig. 2:**
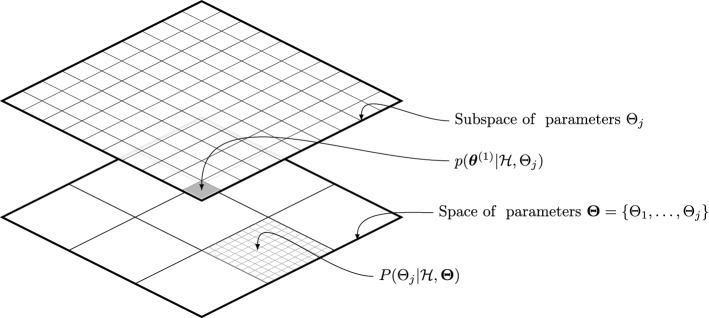
Schematic representation of the proposed two-level Bayesian model assessment framework

Third, since the identification of the model parameters can be computationally challenging when dealing with significantly large search spaces, this paper proposes a novel two-stage inverse problem implementation methodology. A schematic view of this methodology is shown in Fig. 2. By this methodology, the Bayesian assessment of the null hypothesis is first performed across several partitions of the parameter space into various parameter subspaces, and then the problem of parameter identification, which is computationally more demanding, is run over those subspaces with higher relative plausibilities. Consequently, the approach has the advantage of being able to identify (1) the values of the model parameters within a given subspace and also (2) the subspaces within the overall parameter space where the null hypothesis is more likely to hold, with quantified uncertainty. Upon the implementation of the proposed methodology to a representative case study, new evidence is obtained regarding the suitability of the D–F theory for non-horizontal flow in unconfined layered systems.

The paper is organized as follows: Sect. 2 presents the formulation of the proposed model. In Sect. 3, the proposed Bayesian framework for model assessment is presented. Section 4 is devoted to providing results and discussing the proposed Bayesian framework for model assessment, along with computational details about implementation. In Sect. 5, a practical engineering example about railway track drainage is provided to illustrate the applicability of the model in a real-life scenario. Finally, some concluding remarks are provided in Sect. 6.

## Proposed Model

### Governing Equations

Let us consider a physical system represented by a two-dimensional *n*-layered sloping porous medium. This system may represent in practice a layered soil overlying an impermeable bed that rises to a peak between drains at the downstream boundaries of the system with water head $H_{B}$, as depicted in Fig. 1. A uniform steady-state vertical recharge flow rate *q* (e.g. precipitation intensity) is considered as an input to the system.

Using the principle of mass conservation (Bear 1972) as point of departure and assuming a laminar flow parallel to the sloping bed, the water flow can be obtained as a function of the horizontal distance *x* as follows:

1 $$\begin{aligned} \sum _{\ell =1}^{n}Q_{\ell }(x)=q\cdot x, \end{aligned}$$

where *n* is the number of *wet layers*, i.e. those laying totally or partially under the water table, and $Q_{\ell }$ is the water flow through the $\ell$th layer at section *x*. This flow can be expressed as $Q_{\ell }=v_{\ell }S_{\ell }$, with $v_{\ell }$ being the velocity of flow (averaged through the thickness) within layer $\ell$ and $S_{\ell }$ the section perpendicular to the flow in that layer. Therefore, Eq. (1) becomes:

2 $$\begin{aligned} \sum _{\ell =1}^{n}S_{\ell }(x)v_{\ell }(x)=q\cdot x. \end{aligned}$$

Based on simple geometry considerations, the section $S_{\ell }(x)$ can be obtained from Fig. 1 as

3 $$\begin{aligned} S_{\ell }(x) = {\left\{ \begin{array}{ll} t_{\ell }(x)\cos \alpha _0 &{} \quad \text {if } \ell < n\\ \left( h(x)-(L_{x}-x)\tan \alpha _{0}-\sum _{i=1}^{n-1}t_{i}(x) \right) \cos \alpha _0 &{} \quad \text {if } \ell = n\\ \end{array}\right. } \end{aligned}$$

where *h*(*x*) is the water table height as a function of the distance *x*, $t_\ell (x)$ is the vertical thickness of the $\ell$th layer at a distance *x*, $L_{x}$ is the horizontal length of the system, and $\tan \alpha _0$ is the slope of the impervious base, as shown in Fig. 1. From Darcy’s law, the velocity of flow within the $\ell$th layer, $\ell =1,\dots ,n$, can be expressed as

4 $$\begin{aligned} v_{\ell }(x)=-K_{\ell }\frac{\hbox {d}h(x)}{\hbox {d}s}, \end{aligned}$$

where $K_{\ell }$ is the hydraulic conductivity of the layer. By substitution of Eqs. (3) and (4) into Eq. (2), the governing equation for unconfined flow in a *n*-layered sloping system is obtained as:

5 $$\begin{aligned} qx\cot \alpha _0=-\frac{\hbox {d}h(x)}{\hbox {d}s}\left( \sum _{\ell =1}^{n-1}K_{\ell }t_{\ell }(x)+K_{n}\Big (h(x)-\sum _{\ell =1}^{n}t_{\ell }(x)-(L_{x}-x)\tan \alpha _0\Big )\right) . \end{aligned}$$

The last differential equation involves two independent variables, namely *x* and *s*, where *s* is a coordinate measured along the sloping bed with $s = 0$ corresponding to the water table height at $x = 0$, and $s=s_{B}$ at $x=L_{x}$. These independent variables can be shown to be geometrically related as (Youngs and Rushton 2009b):

6 $$\begin{aligned} s_{B}-s=\frac{L_{x}-x}{\cos \alpha _0}+\big (h(x)-(L_{x}-x)\tan \alpha _0\big )\sin \alpha _0. \end{aligned}$$

Next, from the chain rule of derivatives:

7 $$\begin{aligned} \frac{\hbox {d}h}{\hbox {d}s}=\frac{\hbox {d}h}{\hbox {d}x}\frac{\hbox {d}x}{\hbox {d}s}, \end{aligned}$$

where ${\mathrm{d}x{/}\mathrm{d}s}$ can be obtained from Eq. (6) as

8 $$\begin{aligned} \frac{\hbox {d}x}{\hbox {d}s}=\left( \cos \alpha _0-\frac{\hbox {d}h}{\hbox {d}x}\sin \alpha _0\right) ^{-1}. \end{aligned}$$

Substituting Eq. (8) into Eq. (7), and then the obtained result in Eq. (5), the differential equation in (5) can be rewritten after some algebraic manipulation as:

9 $$\begin{aligned} qx=-\phi (x)K_{n}\frac{\hbox {d}h}{\hbox {d}x}, \end{aligned}$$

where

10 $$\begin{aligned} \phi (x)=h(x)-L_{x}\tan \alpha _0+\sum _{\ell =1}^{n-1}\left( \frac{K_{\ell }}{K_n}-1\right) t_{\ell }(x)+\left( 1-\frac{q}{K_n}\right) x\tan \alpha _0. \end{aligned}$$

Note that the governing equation in (9) assumes that groundwater velocity is constant through each layer at every cross section perpendicular to the impervious base, so the whole flow is considered as a set of *streamtubes* parallel to the base. In addition, no-flow and constant head boundary conditions are assumed at the left-hand and downstream boundaries, respectively; thus:

11a $$\begin{aligned} \frac{\hbox {d}h}{\hbox {d}x}\bigg |_{x=0}&=0, \end{aligned}$$

11b $$\begin{aligned} h(x_{B})&=H_{B}, \end{aligned}$$

where $x_{B}$ is the abscissa of the downstream boundary with known water head $H_{B}$.

### Solution Method

The expression in Eq. (9) together with the boundary conditions in (11) constitutes a nonlinear first-order differential equation in *h*(*x*) with no closed-form explicit solution. An implicit parametric solution specified by $\mathscr {W} \ni w \mapsto \left( x(w),h(w)\right) \in \mathbb {R}^{2}$, can be obtained by the variable change:

12 $$\begin{aligned} w=x^{-1} \phi (x), \end{aligned}$$

which allows Eq. (9) to be rewritten as:

13 $$\begin{aligned} \frac{\hbox {d}h}{\hbox {d}x}=-\frac{1}{w}\frac{q}{K_{n}}. \end{aligned}$$

An expression for *x*(*w*) can be obtained by differentiation w.r.t *w* based on Eq. (12), resulting after some algebraic manipulation in:

14 $$\begin{aligned} \frac{\hbox {d}x}{\hbox {d}w}=-\frac{wx}{w^2-\bigg (\left( 1-q/K_n\right) \tan \alpha _0+\sum _{\ell =1}^{n-1}\left( K_{\ell }/K_n-1\right) t_{\ell }^{'}(x)\bigg )w+q/K_n}. \end{aligned}$$

Note that when the thickness of the $\ell$th layer $t_{\ell }(x)$ is a linear function, then $t_{\ell }^{'}(x)=\hbox {d}t_{\ell }(x)/\hbox {d}x$ is a constant given by $t_{\ell }^{'}(x)=\tan \alpha _{\ell -1}-\tan \alpha _{\ell }$, $\ell =1,\ldots , n$, where $\tan \alpha _{\ell }$ is the slope of the upper boundary of the $\ell$th layer. Therefore, the differential equation in (14) becomes a first-order linear differential equation of the form:

15 $$\begin{aligned} \frac{\hbox {d}x}{\hbox {d}w}+xf(w)=0, \end{aligned}$$

where $f(w)=w/(w^2+bw+c)$ and *b*, *c* are constants defined as follows:

16a $$\begin{aligned} b&=-\left( 1- \frac{q}{K_n}\right) \tan \alpha _0-\sum _{\ell =1}^{n-1}\left( \frac{K_{\ell }}{K_n}-1\right) (\tan \alpha _{\ell -1}-\tan \alpha _{\ell }), \end{aligned}$$

16b $$\begin{aligned} c&=\frac{q}{K_n}. \end{aligned}$$

The differential equation in (15) can now be solved using the technique of separating variables; thus:

17 $$\begin{aligned} x(w)=x_{B}\exp \left( -\int _{w_B}^{w} f(\zeta )\hbox {d}\zeta \right) , \end{aligned}$$

where $\zeta$ is a dummy variable for integration and $w_B$ is obtained as $w_B=w(x_B)$, using the expression for the variable change given in Eq. (12). The integral in (17) is known in closed form, given by

18 $$\begin{aligned} F(\zeta )=\int f(\zeta )\hbox {d}t=\frac{1}{2}\ln \left( \zeta ^2+b\zeta +c\right) -\frac{b}{\sqrt{4c-b^2}}\hbox {arctan}\frac{2\zeta +b}{\sqrt{4c-b^2}} \end{aligned}$$

with $4c-b^2>0$. Therefore, Eq. (17) simplifies to:

19 $$\begin{aligned} x(w)=x_B\exp \big (F(w_B)-F(w)\big ), \end{aligned}$$

where parameter *w* is defined within the subspace $\mathscr {W}=[w_B,\infty )\subset \mathbb {R}^{+}$. By taking values $w \in \mathscr {W}$, (e.g. by defining a grid within $\mathscr {W}$), values for *x*(*w*) can be readily obtained from Eq. (19), which are subsequently used to obtain values for the water table height *h*(*w*) from Eq. (10), as:

20 $$\begin{aligned} h(w)=\underbrace{w x(w)}_{\phi }+L_x\tan \alpha -\sum _{\ell =1}^{n-1}\left( \frac{K_{\ell }}{K_n}-1\right) t_{\ell }(x)-\left( 1-\frac{q}{K_n}\right) x(w)\tan \alpha _0. \end{aligned}$$

It should be noted that the proposed solution in Eq. (20) assumes that the water table is wholly contained within the *n*th layer, i.e. $\sum _{\ell =1}^{n-1}t_{\ell }(x)\leqslant h(x)<\sum _{\ell =1}^{n}t_{\ell }(x),~\forall x \in (0,x_{B}]\subset \mathbb {R}^{+}$. However, this is a particular case of a more general one where the water table may cross the boundary between layers with different hydraulic conductivities at an unknown point $x_C\in (0,x_{B}]$. In this case, the complete solution for the water table will be given by a piecewise continuous function where each sub-function is defined in the generic interval $(x_C,x_B]$, with $x_B$ being the abscissa of the known boundary condition, and $x_C$ the abscissa of the crossing point, which becomes the known boundary condition for the subsequent sub-function. The determination of $x_C$ may be challenging especially when considering layers of contrasting hydraulic conductivities (Youngs and Rushton 2009b). A generic trial and error method might be adopted to approximate $x_C$, although this method may lead to error propagation that is hard to control. To overcome this drawback, the Newton–Raphson method (Carnahan 1969) is applied here to systematically obtain a parametric approximation to the abscissa $x_{C}$ with a controlled level of accuracy. To this end, let us define the function

21 $$\begin{aligned} \delta (w)=h(w)-z_{n}(w) \end{aligned}$$

as the difference between the water head *h* (given by Eq. (20)) and the vertical height of the boundaries of layer *n*, $z_n$, which is defined by:

22 $$\begin{aligned} z_{n}(w)=(L_x-x(w))\tan \alpha _0+\sum _{\ell =1}^{n^*}t_{\ell }(x(w)) \end{aligned}$$

with $n^*=n-1$ if the water table crosses the lower boundary of the *n*th layer, and $n^*=n$ otherwise. Thus, $x_C=x(w_C)$ can be obtained as the point where $\delta (w_C)=0$ holds. By Newton–Raphson’s formula, an estimation of $w_C$ can be obtained as follows:

23 $$\begin{aligned} w_{C}^{(i+1)}=w_{C}^{(i)}-\frac{\delta (w_{C}^{(i)})}{\delta '(w_{C}^{(i)})}, \end{aligned}$$

where $w_{C}^{(i)}$ denotes the *i*th iterating approximation to $w_C$. The term $\delta (w_{C}^{(i)})$ in Eq. (23) is obtained by Eqs. (20) and (22) as:

24 $$\begin{aligned} \delta (w_{C}^{(i)})=w_{C}^{(i)} x_{C}^{(i)}-\sum _{\ell =1}^{n^*}\frac{K_{\ell }}{K_n}t_{\ell }(x_{C}^{(i)})+\frac{q}{K_n}x_{C}^{(i)}\tan \alpha _0, \end{aligned}$$

where $x_{C}^{(i)}=x(w_{C}^{(i)})$. The derivative $\delta '(w_{C}^{(i)})$ in Eq. (23) can be obtained from the chain rule as follows:

25 $$\begin{aligned} \delta '(w)=\frac{\mathrm{d}x}{\mathrm{d}w}\left( \frac{\mathrm{d}h}{\mathrm{d}x}-\frac{\mathrm{d}z_{n}}{\mathrm{d}x}\right) , \end{aligned}$$

where $\hbox {d}z_n/\hbox {d}x=-\tan \alpha _{n^*}$, and $\hbox {d}h/\hbox {d}x$, $\hbox {d}x/\hbox {d}w$ are given by Eqs. (13) and (15), respectively. After some algebraical manipulation, Eq. (25) rewrites as:

26 $$\begin{aligned} \delta '(w_{C}^{(i)}) = x_{C}^{(i)}\frac{q/K_n-w_{C}^{(i)}\tan \alpha _{n^{*}}}{(w_{C}^{(i)})^2+bw_{C}^{(i)}+q/K_n}, \end{aligned}$$

where *b* is given by Eq. (16a). Finally, by substituting expressions (24) and (26) into Eq. (23), an iterative approximation to $w_C$ is obtained starting with an initial value $w_{C}^{(i=0)}$ and repeating the process for increasing values of $i \in \mathbb {N}$ until $|x(w_{C}^{(i)})-x(w_{C}^{(i-1)})|<\epsilon$, with $\epsilon$ being a sufficiently small error tolerance. An algorithmic description of the proposed piecewise prediction of the steady-state unconfined water table in layered porous media is provided in Algorithm 1.
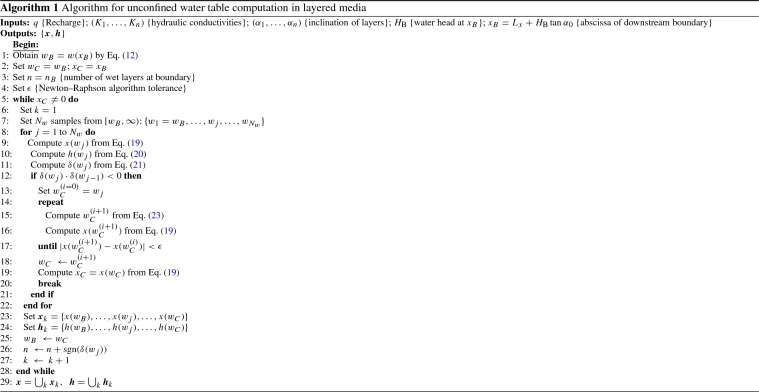


## Bayesian Model Assessment

The model proposed in Sect. 2 is just an idealization of reality based on a set of modelling assumptions. For a particular system output (e.g. water table height), the validity of such simplifying assumptions depends on the adopted values of certain model parameters, such as hydraulic conductivities or slope of layers. A Bayesian inverse problem framework is proposed in this section to efficiently identify the value of the model parameters that better suit the hypothesis that both the proposed model and a reference numerical model given by MODFLOW-NWT (Niswonger et al. 2011) render identical outputs. MODFLOW-NWT is a MODFLOW variant that uses the Newton–Krylov method (Knoll and Keyes 2004) and unstructured, asymmetric matrix solvers to numerically solve the exact formulation of the two-dimensional groundwater flow problem. MODFLOW-NWT is shown to be particularly suitable for unconfined layered systems like the one considered here where the water table crosses the interface between layers with contrasting hydraulic conductivities (Painter et al. 2008; Keating and Zyvoloski 2009). To avoid repetition of the literature, the interested reader is referred to Harbaugh (2005) and Niswonger et al. (2011) for specific details about MODFLOW modelling.

### General Settings

Let $\varvec{f}=\varvec{f}(\varvec{x},\varvec{u};\varvec{\theta })$ be the water table height as given by the proposed model in Sect. 2 for a particular system configuration, where $\varvec{x}=(x_1,\dots ,x_i,\dots ,x_{n_{\varvec{x}}})\in \mathbb {R}^{n_{\varvec{x}}}$ are the abscissa values where $\varvec{f}$ is evaluated, $\varvec{u}\in \mathbb {R}^{n_{\varvec{u}}}$ is a vector containing known model inputs (e.g. geometry inputs), and $\varvec{\theta }\in \varvec{\Theta }\subset \mathbb {R}^{n_{\varvec{\theta }}}$ are model parameters (e.g. hydraulic conductivities) defined over parameter space $\varvec{\Theta }\subset \mathbb {R}^{n_{\varvec{\theta }}}$. Let us also consider a *reference model* for unconfined water table prediction denoted by $\varvec{g}=\varvec{g}(\varvec{x},\varvec{v};\varvec{\theta })$, which, in the absence of experimental data, constitutes our best available knowledge about the system being represented. In this study, $\varvec{g}=\varvec{g}(\varvec{x},\varvec{v};\varvec{\theta })$ will be represented by the solution given by the FD model MODFLOW-NWT, with $\varvec{v}\in \mathbb {R}^{n_{\varvec{v}}}$ being particular model inputs defining the geometry and the configuration of the numerical model.

Observe that aside from model inputs, both models share the same set of model parameters $\varvec{\theta }$, defined over the space $\varvec{\Theta }\subset \mathbb {R}^{n_{\varvec{\theta }}}$. Let us now consider that the hypothesis $\mathscr {H}\triangleq \varvec{f}(\varvec{x},\varvec{u};\varvec{\theta })\equiv \varvec{g}(\varvec{x},\varvec{v};\varvec{\theta })$ arises; i.e. both models are hypothesized to render identical outputs. Thus, the goal is to estimate the extent of agreement with hypothesis $\mathscr {H}$ for the values of model parameters $\varvec{\theta }$ within $\varvec{\Theta }$. This assessment could be carried out by defining a suitable discrepancy function $J(\varvec{\theta })$ such as the $\ell _2$-norm of the difference between the two model outputs as follows:

27 $$\begin{aligned} J(\varvec{\theta })=\left( \sum _{i=1}^{n_{\varvec{x}}} | \varvec{f}(x_i,\varvec{u};\varvec{\theta })-\varvec{g}(x_i,\varvec{v};\varvec{\theta })|^2\right) ^{\frac{1}{2}} \end{aligned}$$

so that $J(\varvec{\theta })$ can be evaluated over a sufficiently fine numerical grid covering the parameter space $\varvec{\Theta }$. However, in large multi-dimensional parameter spaces (e.g. multiple layers with hydraulic conductivities ranging from $10^{-6}$ to $10^{-2}$ m/s), this methodology would require a massive amount of grid points to evaluate Eq. (27), thus leading to a substantial increase in computational complexity. Besides, such a discrepancy function neglects the uncertainty arising from modelling assumptions and errors (discretization error, truncation error, numerical solver adopted for MODFLOW, etc.).

Henceforth, a more principled and efficient method is to assess the *degree of belief* of hypothesis $\mathscr {H}$ for a specific set of model parameters $\varvec{\theta }$, by assuming that $J(\varvec{\theta })$ is uncertain and that it follows a probability model denoted by $p(\mathscr {H}|\varvec{\theta })$. To this end, $J(\varvec{\theta })$ is conservatively assumed to be modelled as a zero-mean Gaussian distribution, i.e. $J(\varvec{\theta })\sim \mathscr {N}(0,\sigma )$, following the principle of maximum information entropy (Jaynes 1957). This principle enables a rational way to establish a probability model for the discrepancy function such that it produces the largest uncertainty (largest Shannon entropy) in the degree of belief of hypothesis $\mathscr {H}$; the selection of any other probability model would lead to an unjustified reduction in such uncertainty (Beck 2010). Thus, the degree of belief of hypothesis $\mathscr {H}$ can be described through the probability model

28 $$\begin{aligned} p(\mathscr {H}|\varvec{\theta })= (2\pi \sigma ^2)^{\frac{-n_{\varvec{x}}}{2}} \exp \left( -\frac{1}{2}\left( \frac{J(\varvec{\theta })}{\sigma }\right) ^2\right) , \end{aligned}$$

where $J(\varvec{\theta })$ is given by Eq. (27). Note that there is not invocation of randomness in Eq. (28). Rather, the probability is interpreted here as a multi-valued propositional logic that expresses the plausibility of hypothesis $\mathscr {H}$ conditioned to models specified by $\varvec{\theta }$. According to Beck (2010), this interpretation of probability is not well known in engineering where there is a widespread belief that probability only applies to aleatory uncertainty (inherent randomness in nature) and not to epistemic uncertainty (degree of belief).

### Assessment of Model Parameters

Equation (28) provides information about the plausibility of the approximate model $\varvec{f}$ reproducing the reference model $\varvec{g}$ for a particular vector of model parameters $\varvec{\theta }$; thus, it represents the *likelihood function* for hypothesis $\mathscr {H}$ given $\varvec{\theta }$. However, our interest precisely lies in the reciprocal information, i.e. to determine the values of $\varvec{\theta }$ among the set of values in $\varvec{\Theta }\subset \mathbb {R}^{n_{\varvec{\theta }}}$ that lead to models that more likely satisfy hypothesis $\mathscr {H}$. This *inverse problem* can be formulated by Bayes’ theorem (Tarantola 2005; Rus et al. 2016), as:

29 $$\begin{aligned} p(\varvec{\theta }|\mathscr {H})=\kappa ^{-1}p(\mathscr {H}|\varvec{\theta })p(\varvec{\theta }), \end{aligned}$$

where $\kappa$ is a normalizing constant defined so that:

30 $$\begin{aligned} \int _{\varvec{\Theta }}p(\varvec{\theta }|\mathscr {H})\hbox {d}\varvec{\theta }=\kappa ^{-1}\int _{\varvec{\Theta }}p(\mathscr {H}|\varvec{\theta })p(\varvec{\theta })\hbox {d}\varvec{\theta }=1. \end{aligned}$$

The term $p(\varvec{\theta })$ in Eq. (29) is a PDF denoting our *prior* degree of belief about the models specified by $\varvec{\theta }$ in regards to the fulfilment of hypothesis $\mathscr {H}$. In this work, the uniform PDF is conservatively adopted for $p(\varvec{\theta })$ as a way of representing our prior state of ignorance about the values $\varvec{\theta }\in \varvec{\Theta }$ satisfying hypothesis $\mathscr {H}$. Note that Bayes’ theorem takes this initial degree of belief and updates it by using the information given by the likelihood function in Eq. (28). The resulting information $p(\varvec{\theta }|\mathscr {H})$ is formally referred to as the *posterior* PDF of model parameters.

In most practical situations, the normalizing constant $\kappa$ in Eq. (28) cannot be evaluated analytically nor readily calculated using numerical integration methods, if the dimension $n_{\varvec{\theta }}$ is not small. Hence, Markov chain Monte Carlo (MCMC) methods (Gilks et al. 1996; Gamerman and Lopes 2006) are commonly used to draw samples from the required PDF in Eq. (29) while circumventing the evaluation of $\kappa$. Among these methods, the Metropolis–Hastings (M–H) algorithm (Metropolis et al. 1953; Hastings 1970) is widely used due to its versatility and simplicity of implementation. The M–H algorithm generates samples from a specially constructed Markov chain whose stationary distribution is the required PDF $p(\varvec{\theta }|\mathscr {H})$. By sampling a candidate model parameter $\varvec{\theta }^{'}$ from a suitably defined *proposal distribution*$\pi (\varvec{\theta }^{'}|\varvec{\theta }^{(\zeta )})$, the M–H algorithm obtains the state of the chain at $\zeta +1$, given the state at $\zeta$, specified by $\varvec{\theta }^{(\zeta )}$. The candidate parameter $\varvec{\theta }^{'}$ is accepted (i.e. $\varvec{\theta }^{(\zeta +1)}=\varvec{\theta }^{'}$) with probability $\text {min}\{1,r\}$ and rejected (i.e. $\varvec{\theta }^{(\zeta +1)}=\varvec{\theta }^{(\zeta )}$) with the remaining probability $1-\text {min}\{1,r\}$, where:

31 $$\begin{aligned} r=\frac{p(\mathscr {H}|\varvec{\theta }^{'})p(\varvec{\theta }^{'})\pi (\varvec{\theta }^{(\zeta -1)}|\varvec{\theta }^{'})}{p(\mathscr {H}|\varvec{\theta }^{(\zeta -1)})p(\varvec{\theta }^{(\zeta -1)})\pi (\varvec{\theta }^{'}|\varvec{\theta }^{(\zeta -1)})}. \end{aligned}$$

The process is repeated until a sufficient amount of samples have been generated so that the monitored acceptance rate (ratio between accepted M–H samples over total amount of samples) reaches an asymptotic behaviour. The reader is referred to Chiachío et al. (2015, 2017) for a pseudocode implementation of M–H algorithm in the context of Bayesian model parameter estimation.

### Assessment of Parameter Subspaces

From a theoretical point of view, the information stemming from Eq. (29) would be enough to identify the values $\varvec{\theta }\in \varvec{\Theta }$ that make hypothesis $\mathscr {H}$ more likely to become true. However, in real-life applications, $\varvec{\theta }$ may take values over a large parameter space $\varvec{\Theta }$ (e.g. hydraulic conductivities ranging from $10^{-10}$ to $10^{-1}$ m/s). In this context, very long chains of samples are expected when using MCMC methods to obtain the posterior PDF of model parameters $p(\varvec{\theta }|\mathscr {H})$ as described in Sect. 3.2, thus leading to a heavy computational burden. A proposed method to overcome this problem is to split $\varvec{\Theta }$ into a set of $n_{s}$ subspaces $\left\{ \Theta _1,\dots ,\Theta _j,\dots ,\Theta _{n_{s}}\right\}$ such that $\varvec{\Theta }=\bigcup _{j=1}^{n_{s}}\Theta _{j}$, and then solve Eq. (29) within those subspaces with higher relative plausibilities to fulfil hypothesis $\mathscr {H}$. This leads to a two-stage Bayesian inverse problem, as depicted in Fig. 2, where first the $n_{s}$ subspaces are ranked according to their overall probability of satisfying the referred hypothesis $\mathscr {H}$, and then, the posterior PDF of model parameters is obtained for a particular subspace $\Theta _j \subset \varvec{\Theta }$, as

32 $$\begin{aligned} p(\varvec{\theta }|\mathscr {H},\Theta _j)=\kappa ^{-1}p(\mathscr {H}|\varvec{\theta },\Theta _j)p(\varvec{\theta }|\Theta _j), \end{aligned}$$

where the conditioning upon $\Theta _j$ is given by:

33 $$\begin{aligned} p(\cdot |\Theta _j) = {\left\{ \begin{array}{ll} \kappa _1p(\cdot ), &{} \quad \text {if } \varvec{\theta }\in \Theta _j \\ 0, &{} \quad \text {otherwise}\\ \end{array}\right. } \end{aligned}$$

with $\kappa _1$ being a normalizing constant.

To obtain the *posterior plausibility* of the *j*th subspace in $\varvec{\Theta }$, i.e.: $P(\Theta _{j}|\mathscr {H},\varvec{\Theta })$, Bayes’ theorem is extended at the level of the subspaces as follows:^1^

34 $$\begin{aligned} P(\Theta _{j}|\mathscr {H},\varvec{\Theta })=\kappa _{2}^{-1}p(\mathscr {H}|\Theta _{j})P(\Theta _{j}|\varvec{\Theta }), \end{aligned}$$

where $\kappa _2$ is a normalizing constant satisfying $\kappa _{2}^{-1}\sum _{j=1}^{n_{s}}p(\mathscr {H}|\Theta _{j})P(\Theta _{j}|\varvec{\Theta })=1$, and $P(\Theta _{j}|\varvec{\Theta })$ is the prior plausibility of the *j*th subspace in $\varvec{\Theta }$, so that $\sum _{j=1}^{n_{s}}P(\Theta _{j}|\varvec{\Theta })=1$. This prior plausibility expresses the initial relative degree of belief of the models evaluated in $\Theta _{j}$ within $\varvec{\Theta }$ in regard to the fulfilment of hypothesis $\mathscr {H}$. The factor $p(\mathscr {H}|\Theta _{j})$ is the *evidence* for model subspace $\Theta _{j}\in \varvec{\Theta }$ and expresses how likely hypothesis $\mathscr {H}$ is satisfied if model parameters in subspace $\Theta _{j}$ are adopted. This evidence can be obtained by using the total probability theorem:

35 $$\begin{aligned} p(\mathscr {H}|\Theta _{j})=\int _{\Theta _j} \underbrace{p(\mathscr {H}|\varvec{\theta },\Theta _{j})}_{\text {Eq.}~(28)}p(\varvec{\theta }|\Theta _{j})\hbox {d}\varvec{\theta }, \end{aligned}$$

where $p(\mathscr {H}|\varvec{\theta },\Theta _{j})$ and $p(\varvec{\theta }|\Theta _{j})$ are the likelihood function and the prior PDF of model parameters $\varvec{\theta }\in \Theta _j$, respectively. Note that the evaluation of the multi-dimensional integral in Eq. (35) is non-trivial except for some particular cases where Laplace’s method of asymptotic approximation can be used (Yuen 2010). One straightforward way to approximate the evidence that is adopted in this paper is by considering the probability integral in Eq. (35) as a mathematical expectation of the likelihood $p(\mathscr {H}|\varvec{\theta },\Theta _{j})$ with respect to the prior $p(\varvec{\theta }|\Theta _{j})$. This approach leads to the direct Monte Carlo method as follows:

36 $$\begin{aligned} p(\mathscr {H}|\Theta _{j})\approx \frac{1}{T_{s}}\sum _{k=1}^{T_{s}}p(\mathscr {H}|\varvec{\theta }^{(k)},\Theta _{j}), \end{aligned}$$

where the $\varvec{\theta }^{(k)}$ are $T_{s}$ samples drawn from the prior $p(\varvec{\theta }|\Theta _{j})$.

## Numerical Validation by Bayesian Assessment

In this section, the proposed model for unconfined water flow in sloping layered porous media is tested using a three-layered unconfined system as an illustrative example. Sections 4.1 and 4.2 describe the system configuration and modelling details for the proposed Bayesian assessment methodology. The results are provided and discussed in Sect. 4.3.

**Fig. 3:**
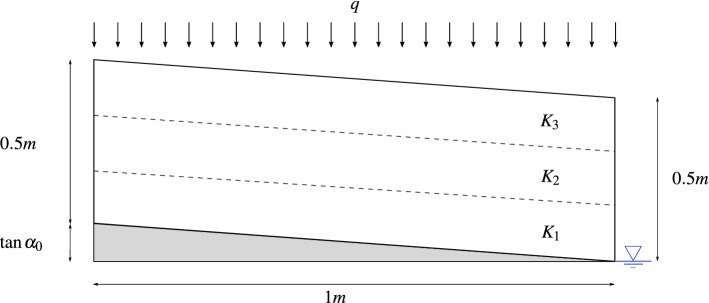
Representative three-layered unconfined aquifer considered as a case study for Bayesian model assessment

### System Configuration

The outcomes of the two-stage Bayesian model assessment methodology presented in Sect. 3 are shown here for the representative system depicted in Fig. 3. In this example, a 1-m-half-width 0.5-m-thick three-layered unconfined sloping porous media is considered. For the sake of illustration simplicity, layers are assumed to remain parallel to the impervious base, i.e. $\tan \alpha _\ell =\tan \alpha _0,~\ell =1,2,3$. On top of the resulting layered system, a 1-m-thick extra layer is added to prevent the water table from reaching the top of the model; however, this upper layer is not considered in the Bayesian assessment. The water head at the downstream boundary is conservatively set to $H_\mathrm{B}=0$. Any other choice for $H_\mathrm{B}$ would lead to better agreements between the proposed model and MODFLOW in the vicinity of the downstream boundary (Youngs and Rushton 2009b). Here, the non-dimensional recharge rate to conductivity ratio of the layers $q/K_\ell$ along with the slope of the impervious base is chosen as the testing parameters, i.e. $\varvec{\theta }=\left\{ {q}/{K_1},{q}/{K_2},{q}/{K_3},\tan \alpha _0 \right\}$. The parameter space $\varvec{\Theta }\subset \mathbb {R}^{4}$ for this system configuration is conveniently partitioned into the subspaces $\{\Theta _1,\Theta _2,\Theta _3,\Theta _4\}$, as specified in Table 1. Note that this splitting may cover in practice recharge rates corresponding to rainfalls with 2–20-year return period (e.g. for a town in the Midlands of England) for porous material ranging from very fine sands to well-sorted sands and gravels. However, the proposed Bayesian model assessment methodology is general, and therefore, the space and subspaces of parameters to be tested can be conveniently defined to cover other validation scenarios.

**Table 1:** Range of values for model parameters defining each subspace

Parameter	$\Theta _{1}$	$\Theta _{2}$	$\Theta _{3}$	$\Theta _{4}$
$q/K_\ell$	$\left[ 0.01,0.015 \right)$	$\left[ 0.015,0.15 \right)$	$\left[ 0.15,1 \right)$	$\left[ 1,1.5 \right]$
$\tan \alpha _0$	$\left[ 0 ,0.5 \right]$	$\left[ 0 ,0.5 \right]$	$\left[ 0 ,0.5 \right]$	$\left[ 0 ,0.5 \right]$

From this standpoint, both the proposed model and the numerical reference model given by MODFLOW-NWT are repetitively run for different values of model parameters $$\varvec{\theta }\in \Theta _{j}\subset \varvec{\Theta },~j=1,\dots ,4$$, driven by the M–H algorithm, so as to obtain the required posterior probabilities in Eq. (29). For the MODFLOW-NWT simulation, a spatial grid is considered by discretizing each model layer into one row and 200 columns. Such discretization is appropriately selected after a grid convergence study such that the solution given by MODFLOW-NWT is independent of the grid size for each subspace of parameters $$\Theta _j \in \varvec{\Theta },~j=1,\ldots ,4$$. The orthomin/stabilized conjugate-gradient solver, also called $$\chi $$MD (Niswonger et al. 2011), is chosen as matrix solver for MODFLOW-NWT. Default solver input values are scaled as suggested by Niswonger et al. (2011) under the “complex” solver option. No-flow boundaries are set at the left-hand boundary ($$x=0)$$ and the impermeable base, while a specified flow boundary is applied to the top of the upper layer using the “Recharge” package (McDonald and Harbaugh 1988). For this example, a constant value $$q=1\times 10^{-5}$$ m/s is adopted for the recharge flux rate; therefore, the variability in $$q/K_\ell ,~\ell = 1,2,3$$, is achieved by correspondingly varying the values for $$K_\ell $$. A head-dependent boundary condition is considered at the right-hand boundary using the “General Head Boundary” package, allowing for *seepage face* formation (Rushton and Youngs 2010; Bear 1972). Water flow across this boundary is obtained from Darcy’s law using a gradient calculated as the difference between the specified head outside the boundary ($$H_\mathrm{B}$$) and the head computed by MODFLOW-NWT on the boundary. To assess the accuracy of the numerical solution, the water budget error (i.e. the difference between water inflow and outflow) is computed for each simulation so that the solution is accepted only if water budget error is less than $$0.5\%$$ (Anderson et al. 2015).

### M–H Algorithm Implementation

As stated in Sect. 3.2, the prior PDF associated with the set of model parameters $\varvec{\theta }$ for a particular subspace $\Theta _j$ is modelled as a uniform distribution defined within the interval of definition of such parameters; i.e. for the *i*th component $\theta _i \in \varvec{\theta }\in \Theta _j$,  $p(\theta _{i}|\Theta _j)=\mathscr {U}(\theta _{i,\min },\theta _{i,\max }),~j=1,\dots ,4$, where $\theta _{i,\min }$ and $\theta _{i,\max }$ are given in Table 1. It should be noted that each component $\theta _i \in \varvec{\theta },~i=1,\dots ,n_{\varvec{\theta }}$, is conservatively assumed to be stochastically independent (Chiachío et al. 2015); thus, $p(\varvec{\theta }|\Theta _j)$ is defined as the unconditional product of the individual priors, i.e. $p(\varvec{\theta }|\Theta _j)=\prod _{i=1}^{n_{\varvec{\theta }}}p(\theta _{i}|\Theta _j),~n_{\varvec{\theta }} = 4,~j=1,\dots ,4$. At the level of the subspaces of parameters, a discrete uniform distribution function is adopted for the prior probabilities of such subspaces, i.e. $P(\Theta _{j}|\varvec{\Theta })={1}/{4}, j = 1,\ldots ,4$, representing our initial state of ignorance about the subspaces where hypothesis $\mathscr {H}$ is more likely to hold. To obtain the required posterior PDF of parameters $p(\varvec{\theta }|\mathscr {H},\Theta _j)$ for the *j*th subspace, the M–H algorithm is applied with a multivariate Gaussian for the proposal PDF, i.e. $\pi (\cdot |\varvec{\theta }^{(\zeta )})=\mathscr {N}(\varvec{\theta }^{(\zeta )},\varvec{\varSigma }_{\pi })$ in Eq. (31), where $\varvec{\varSigma }_{\pi } \in \mathbb {R}^{n_{\varvec{\theta }}\times n_{\varvec{\theta }}}$ is the covariance matrix of the random walk. Note that since model parameters $\varvec{\theta }$ are assumed to be stochastically independent, $\varvec{\varSigma }_{\pi }$ is a diagonal matrix, i.e. $\varvec{\varSigma }_{\pi }=\hbox {diag}(\sigma _{\pi ,1}^{2},\ldots ,\sigma _{\pi ,n_{\varvec{\theta }}}^{2})$; hence, each component parameter in $\varvec{\theta }$ performs an independent random walk within $\Theta _j$. The diagonal elements of $\varvec{\varSigma }_{\pi }$ are appropriately selected through initial test runs such that the monitored acceptance rate (ratio between accepted M–H samples over total amount of samples) is within the suggested range $\left[ 0.2,0.4\right]$ for the M–H algorithm (Roberts and Rosenthal 2001). For the definition of Eq. (28), the standard deviation of the discrepancy function is set to $\sigma =0.05$, taking it as known. This parameter has been shown to have a relatively low influence on the model parameters identification.

### Results and Discussion

**Fig. 4:**
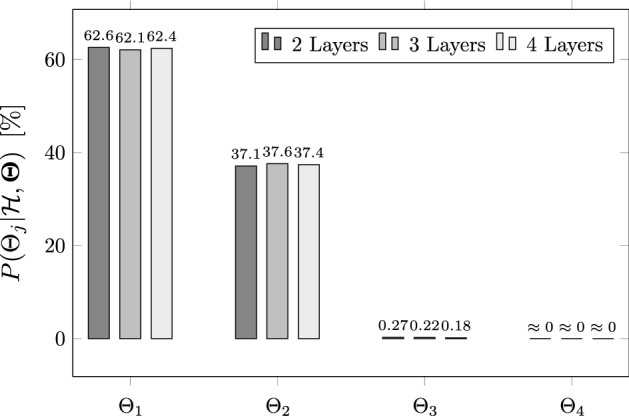
Posterior relative plausibilities of parameter subspaces $\Theta _1$ to $\Theta _4$ for representative system shown in Fig. 3 with two, three and four layers

The assessment and rank of the various subspaces of parameters defined in Table 1 is shown in Fig. 4 based on the posterior plausibilities $P(\Theta _j|\mathscr {H},\varvec{\Theta }),~j=1,\dots ,4$. Note that, in addition to the three-layered porous media considered in this case study, a variation of the system configuration consisting of splitting the 0.5-m-thick porous material into two and four equal-thickness layers is considered as a way to provide insight about the influence of the number of layers on the plausibility of those subspaces. As evident from the results, subspaces $\Theta _1$ and $\Theta _2$ accumulate almost all of the probability mass, with $\Theta _1$ yielding more than $60\%$ of the relative plausibility. This information allows us to identify in advance the subspaces in $\varvec{\Theta }$ where hypothesis $\mathscr {H}$ is more likely to hold, before running a full Bayesian inverse problem at the level of the parameters by MCMC simulation. Observe also that the posterior plausibilities in Fig. 4 are insensitive to the number of layers, at least for the system considered in this study.

**Fig. 5:**
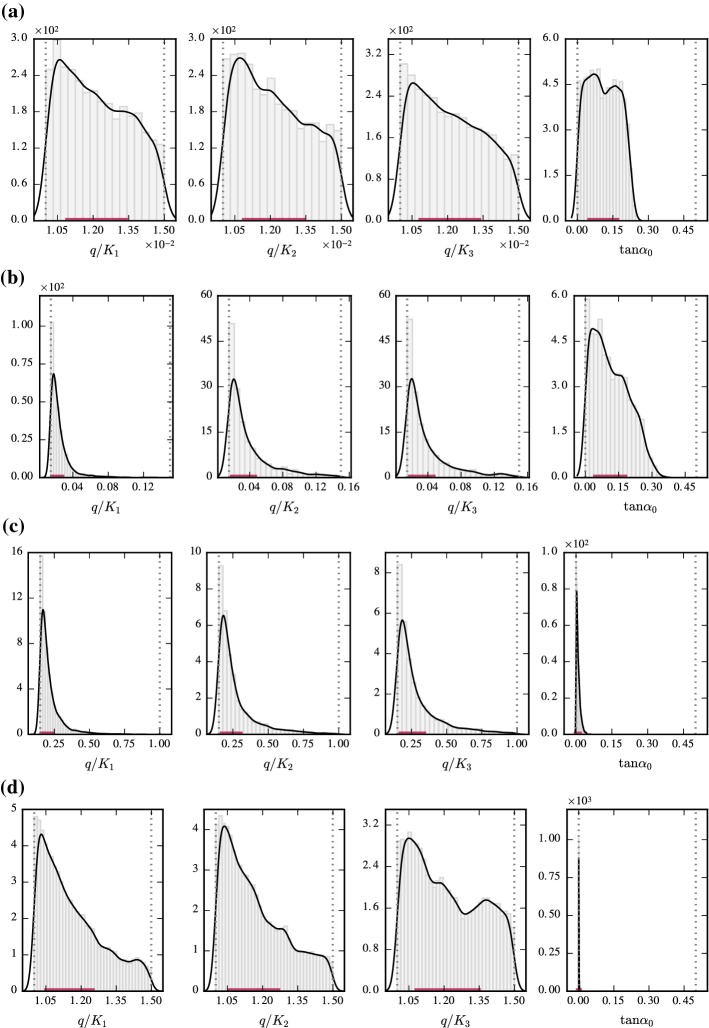
Kernel density estimates of the marginal posteriors $p(\theta _i|\mathscr {H},\Theta _j),~i=1,\ldots ,4$ for subspaces $\Theta _{1}$ to $\Theta _{4}$, shown in panels **a**–**d**, respectively. The grey-dotted vertical lines are to represent the limiting values for the uniform prior PDFs. The red solid line on the horizontal axis denotes the [25–75%] probability band of the marginal posterior PDFs, denoting the subregions where hypothesis $\mathscr {H}$ is more likely to become true

At the level of the model parameters, Fig. 5 shows the results for the Bayesian model parameter assessment for the different subspaces defined in Table 1. As apparent from the results, the proposed Bayesian methodology is able to identify through probability densities the subregion within the space of parameters where the hypothesis $\mathscr {H}$ of model similarity is more likely to hold. Observe from Fig. 5a, b that the proposed model is likely to provide accurate predictions of the water table even for slopes up to $30\%$ for subspaces $\Theta _1$ and $\Theta _2$. These subspaces, which were indicated to be the most plausible following the Bayesian model assessment at the subspace level (recall Fig. 4), include values for $q/K_\ell$ within the non-dimensional range $\left[ 0.015, 0.15\right)$, which covers a significant part of practical groundwater problems in science and engineering. These results provide new evidence against the general notion that D–F theory must be restricted to regions with a small inclination of the water table and where the vertical flow component may be neglected. Instead, the vertical recharge to hydraulic conductivity ratio (*q* / *K*) formally emerges as a critical parameter for model assessment so that when conveniently bounded, the assumption of unidimensional flow parallel to the impervious base can be safely adopted. For subspaces $\Theta _3$ and $\Theta _4$, the posterior range of plausible values for the slope parameter is significantly reduced with respect to the prior around the value $\tan \alpha _0=0$, as evident from Fig. 5c, d. This is also manifested in the lower values for the relative plausibilities for those subspaces, as shown in Fig. 4. These low values for the plausibilities can be explained based on the likelihood function, which is evaluated using prior samples from a region of the parameter subspace far from the narrow region of high likelihood (recall Eq. 35), which requires values of the slope parameter close to $\tan \alpha _0=0$ (horizontal flow).

**Fig. 6:**
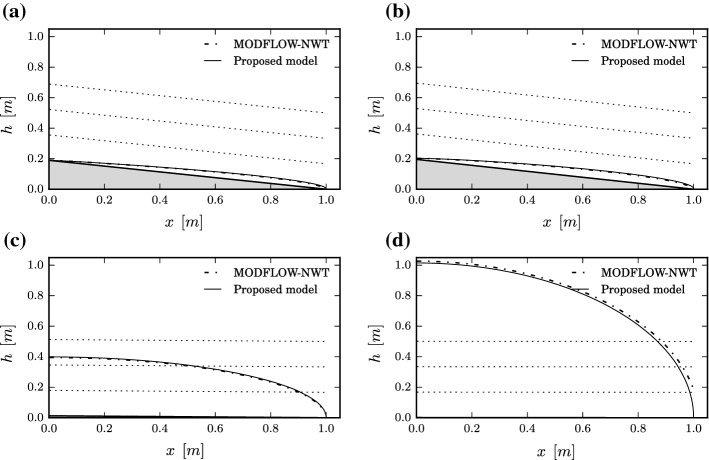
Water table heights as predicted by the proposed model and MODFLOW-NWT model for the system depicted in Fig. 3 using a representative value for $\varvec{\theta }$ taken at a median absolute deviation of the posterior median. The results are shown in panels **a**–**d** for subspaces $\Theta _{1}$ to $\Theta _{4}$, respectively. **a**$\Theta _{1}$, **b**$\Theta _{2}$, **c**$\Theta _{3}$, **d**$\Theta _{4}$

Finally, as a proof of model fitting accuracy, a simulation experiment using the outputs of both models, namely the MODFLOW-NWT model and the model proposed here, has been carried out and the results are shown in Fig. 6. To prevent the models from being evaluated for the most plausible parameters in terms of fulfilment of hypothesis $\mathscr {H}$, model outputs are conservatively simulated using a representative value for model parameters $\varvec{\theta }=\hat{\varvec{\theta }}$ taken at a median absolute deviation distance from the posterior median, i.e. $\hat{\varvec{\theta }}=\mathrm {median}(\varvec{\theta }) + \mathrm {median}(|\varvec{\theta }-\mathrm {median}(\varvec{\theta })|)$, where the median is estimated using samples from the posterior PDF of model parameters $p(\varvec{\theta }|\mathscr {H},\Theta _j)$, $j=1,\dots ,4$. Observe the marked general agreement even for subspaces $\Theta _3$ and $\Theta _4$ which show very low relative posterior plausibilities. A local exception to such goodness of fit can be observed in the vicinity of the downstream face, where the adequacy of the assumed hypothesis of flow parallel to the base is noticeably poor, due to the formation of a seepage face. This seepage face is properly simulated by MODFLOW-NWT, but cannot be predicted by the proposed model due to the assumed hypothesis of unidimensional flow (Bear 1972). Notwithstanding, some *ad hoc* solutions have been suggested in the literature to emulate the effect of the seepage face on the D–F solution by introducing an artificial boundary condition (Mizumura 2009; Rushton and Youngs 2010; Dan et al. 2012). These solutions could be conveniently incorporated into the proposed model to better fit the reality in such region.

## Engineering Case Study

**Fig. 7:**
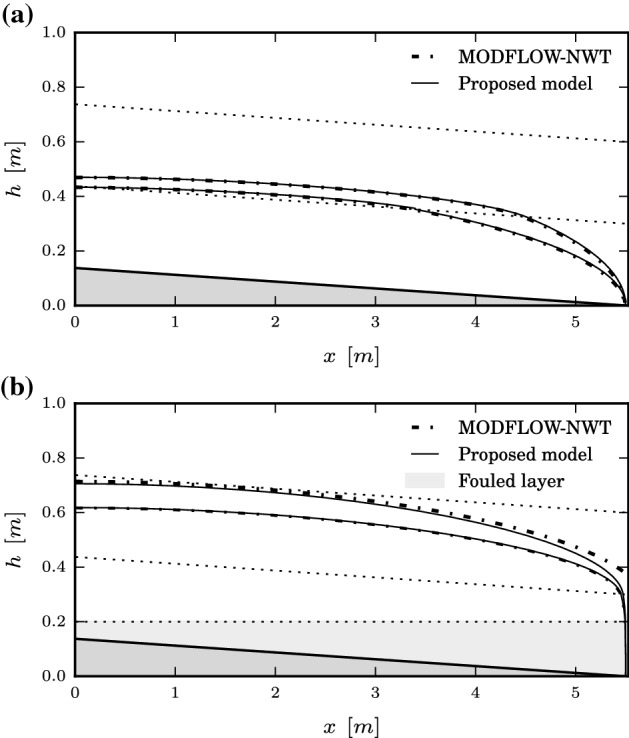
Steady-state water table prediction using both the proposed model and MODFLOW-NWT for a 5.5-m-half-width track section for two scenarios of ballast degradation. Lower water tables correspond to $q=3\times 10^{-6}$ m/s, and upper ones to $q=5\times 10^{-6}$ m/s. Panel (a): $t_{\ell }=0.3$ m, $\ell =1,2$; $\tan \alpha _{\ell }=0.025,~\ell =0,1,2$; $\{K_1=0.5,~K_2=5\}\times 10^{-3}$ m/s. Panel (b): $\tan \alpha _{\ell }=0.025,~{\ell }=0,2,3$, $\tan \alpha _{1}=0$; $\{K_1=0.01,~K_2=0.1,~K_3=1\}\times 10^{-3}$ m/s. **a** Moderate degradation, **b** severe degradation

Railway track drainage plays a significant role on the overall safety and serviceability of the railway infrastructure; however, it has been commonly given insufficient attention during design and operation (Selig and Waters 1994). This is perhaps due to the lack of practical but accurate modelling tools for practitioners and maintenance engineers, along with the complexity of available computational models for numerical simulation. To illustrate the applicability and efficiency of the proposed model in the context of this engineering application, the steady-state water table elevation, as predicted by the proposed model, is computed and compared against MODFLOW-NWT for a system representing a ballasted railway track section (ballast and sub-ballast) under service conditions. To assess the effect of the track degradation on the water table elevation, the models are simulated for two scenarios representing different levels of ballast degradation in terms of loss of hydraulic conductivity. Results and configuration details are shown in Fig. 7 for both scenarios. Two recharge rates $q=\{3\times 10^{-6},5\times 10^{-6} \}$ m/s are considered for each case study, which approximately correspond to the rainfall intensity for 1 hour in the Midlands of England for 2- and 5-year return periods, respectively. Note in Fig. 7b that the severely degraded scenario is simulated by introducing an additional fouled layer at the bottom of the track section. This fouled layer is a consequence of the hydraulic pumping of sub-soil fine particles through the ballast voids, which has been reported by several authors not only for railway tracks (Selig and Waters 1994; Duong et al. 2014), but also for road pavements (Alobaidi and Hoare 1994, 1999; Yuan et al. 2007). Thus, the internal drainage of the track section under severe degradation results in a three-layered unconfined sloping porous medium with non-parallel layers, where no analytical formulation in the current literature is applicable. To simulate both scenarios using MODFLOW-NWT, each layer is discretized into 500 columns and 10 sublayers to allow for seepage formation. Such discretization is assessed through initial test runs such that the solution given by MODFLOW-NWT becomes independent of the grid size. The same boundary conditions and solver input values as those specified in Sect. 4.3 for MODFLOW simulation are adopted; therefore, they are not repeated here. The water head at the downstream boundary is conservatively set to $H_\mathrm{B}=0$ for both moderate and severe degradation cases. In view of Fig. 7, the agreement between the proposed model and MODFLOW-NWT is markedly good for the moderate degradation scenario for both rainfall intensities. For the severely degraded system, the agreement is fairly good except in the vicinity of the downstream boundary, due to the formation of a seepage face, as discussed in Sect. 4.3.

## Conclusions

An approximate model based on the Dupuit–Forchheimer theory has been presented to efficiently predict the steady-state water table height in sloping layered porous media with recharge. The model was developed as an alternative to the numerical modelling version of the same problem, which becomes computationally intractable in a number of practical problems requiring multiple model evaluations. To verify and validate the proposed model, a novel approach based on Bayesian hypothesis testing has been developed to evaluate the accuracy of the new model against the numerical model MODFLOW-NWT, an open-source finite-difference code by the U.S. Geological Survey for unconfined groundwater flow, considered here as reference model. The assessment is carried out through probabilities that measure the relative extent of agreement between both models for the many possible values of model parameters while accounting for the underlying modelling uncertainties. The numerical implementation of this Bayesian methodology is facilitated by considering multiple subspaces within the overall parameter space, and the probabilities across such multiple subspaces are integrated using principles of conditional probability and total probability. The ratio of vertical recharge to hydraulic conductivity formally emerges as a critical parameter for model accuracy so that when conveniently bounded, both the proposed model and MODFLOW-NWT provide almost identical results.

Building on this work, a future research direction is the application of the proposed Bayesian framework to infer an approximate model for unconfined flow in large-scale heterogeneous porous media taking as reference model a stochastic numerical modelling approach (Mantoglou 1992; Mousavi Nezhad et al. 2011), which would allow the consideration of spatially variable hydraulic properties in the assessment. Another desirable further work in the context of model development is the assessment of a sound approach to improve the lack of fitting accuracy of the proposed model in the vicinity of the seepage face.

## Footnotes

1 Note that, while $p(\cdot )$ is used to denote a probability density function (PDF), $P(\cdot )$ is used to denote probability.
